# Quality of Prostate Cancer Treatment Information on Cancer Center Websites

**DOI:** 10.7759/cureus.580

**Published:** 2016-04-20

**Authors:** Caleb Dulaney, Olivia Claire Barrett, Soroush Rais-Bahrami, Daniel Wakefield, John Fiveash, Michael Dobelbower

**Affiliations:** 1 Department of Radiation Oncology, University of Alabama at Birmingham; 2 Department of Urology, University of Alabama at Birmingham; 3 College of Medicine, University of Tennessee Health Sciences Center

**Keywords:** internet, decision making, prostate cancer

## Abstract

**Introduction:**

Cancer center websites are trusted sources of internet information about treatment options for prostate cancer. The quality of information on these websites is unknown. The objective of this study was to evaluate the quality of information on cancer center websites addressing prostate cancer treatment options, outcomes, and toxicity.

**Materials and methods:**

We evaluated the websites of all National Cancer Institute-designated cancer centers to determine if sufficient information was provided to address eleven decision-specific knowledge questions from the validated Early Prostate Cancer Treatment Decision Quality Instrument. We recorded the number of questions addressed, the number of clicks to reach the prostate cancer-specific webpage, evaluation time, and Spanish and mobile accessibility. Correlation between evaluation time and questions addressed were calculated using the Pearson coefficient.

**Results:**

Sixty-three websites were reviewed. Eighty percent had a prostate cancer-specific webpage reached in a median of three clicks. The average evaluation time was 6.5 minutes. Information was available in Spanish on 24% of sites and 59% were mobile friendly. Websites provided sufficient information to address, on average, 19% of questions. No website addressed all questions. Evaluation time correlated with the number of questions addressed (R^2^ = 0.42, p < 0.001).

**Conclusions:**

Cancer center websites provide insufficient information for men with localized prostate cancer due to a lack of information about and direct comparison of specific treatment outcomes and toxicities. Information is also less accessible in Spanish and on mobile devices. These data can be used to improve the quality and accessibility of prostate cancer treatment information on cancer center websites.

## Introduction

Among the key ways of improving cancer care outlined in the Institute of Medicine’s report, “Delivering High Quality Cancer Care: Charting a New Course for a System in Crisis,” are 1) using information technology to provide better information about the benefits of treatments and 2) ensuring patients are engaged and understand their diagnoses so they can make informed decisions [1]. Men with early-stage prostate cancer face considerable challenges in weighing treatment options and making a final decision about management. Standard management options for localized prostate cancer include open and robotic radical prostatectomy, external beam radiation therapy, low dose rate and high dose rate interstitial brachytherapy, and active surveillance. Emerging focal therapies include thermal ablation (cryosurgery, radiofrequency ablation, and thermal ablation), high intensity focused ultrasound (HIFU), and vascular targeted photodynamic (VTP) therapy [2]. With similar efficacy among the standard treatment options, the decision making process can be stressful and confusing and final treatment decisions are often dissonant from treatment goals [3].

Internet resources are important sources of information that are increasingly utilized by men considering treatment options for prostate cancer. Nearly 60% of men consult internet sources of information in making their treatment decision, which is similar to the proportion that discusses options with a second physician [3]. Cancer center and hospital websites are trusted sources of internet information about treatment options for prostate cancer. The quality of treatment information on these sites supporting shared decision making is unknown. The purpose of this study was to evaluate the quality of information addressing early prostate cancer treatment options, outcomes, and toxicity on National Cancer Institute (NCI)-designated cancer center websites using a validated decision quality instrument (DQI).

## Materials and methods

A list of cancer centers for this investigation was obtained from the NCI’s website (www.cancer.gov). The websites of all NCI-designated clinical and comprehensive cancer centers were evaluated to determine if sufficient information was provided to answer each of the eleven decision-specific knowledge questions from the validated Early Prostate Cancer Treatment DQI [4]. Websites were reviewed by two physicians with experience treating prostate cancer. During website evaluation, each reviewer was blinded from the evaluations of the other reviewer. The Early Prostate Cancer Treatment DQI is designed to test patient knowledge about their condition and treatment options, concordance of treatment choices with underlying patient goals, and the extent of patient involvement in the decision making process. Decision-specific knowledge questions within the DQI were generated by a group of prostate cancer experts and patients to represent important information needed to make an informed decision about treatment. 

Websites were generally navigated by clicking links or using the search bar on the homepage to find the prostate cancer-specific webpage if one existed. The search bar could also be used to search for treatment information on the website. All videos and downloadable information were included in the evaluation. Information provided on links to external sites was not included in the evaluation. The number of clicks to reach the prostate-specific webpage and evaluation time were recorded. Evaluation time was considered the time spent evaluating the website after reaching the prostate cancer-specific webpage. For sites with no prostate cancer-specific webpage, evaluation time was 0 minutes.

The prostate-specific webpage was also evaluated to determine if there was an option of viewing information in Spanish. This was done by searching the homepage and prostate cancer-specific webpage for an option to change the language of the text or to view the website in another language. Website reviewers were not fluent in Spanish and the quality of information in Spanish, if available, was not evaluated as part of the study. Mobile device accessibility was determined by using the Google mobile friendly test tool available at https://www.google.com/webmasters/tools/mobile-friendly/. We also recorded the presence of a listing of prostate cancer-specific clinical trials by searching the homepage and prostate cancer-specific webpage for clinical trials. We then determined if an option was available for viewing prostate cancer-specific clinical trials as opposed to a general listing of all clinical trials. We recorded whether or not the prostate cancer-specific webpage listed or had a link to a list of physicians involved in treating prostate cancer at that institution. Inter-observer variability was measured using percent agreement and kappa coefficient. Pearson correlation coefficient was used to calculate the correlation between evaluation time and number of questions addressed.

## Results

Sixty-three NCI-designated clinical and comprehensive cancer center websites were independently evaluated. One was omitted (St. Jude Children’s Research Hospital) due to primary pediatric oncology focus. Eighty percent of websites had a prostate cancer-specific webpage reached in a median of three clicks from the center’s home page (range 1-6). The average website evaluation time was 6.5 minutes. Information was available in Spanish on 24% of sites and 59% were mobile friendly. Eighty-seven percent of websites listed prostate cancer-specific clinical trials and 80% listed the members of the prostate cancer treatment team. Inter-observer variability was moderate with 73% agreement and an average kappa coefficient of 0.34. 

Websites were ranked by the average number of questions addressed. The top five websites are listed in Table 1. Websites provided sufficient information to address, on average, 19% of the DQI questions. No website addressed all questions. Seventeen percent of websites did not have sufficient information to address any of the DQI questions.

**Table 1:** Top 5 NCI clinical and comprehensive cancer center websites ranked by number of decision-specific knowledge questions addressed.

Rank	Cancer Center	Questions Addressed (%)
1	Beth Israel Deaconess Medical Center	73%
2	Stanford University Cancer Institute	59%
2	University of California, San Diego Moores Comprehensive Cancer Center	59%
4	MD Anderson Cancer Center	55%
5	Mayo Clinic Cancer Center	50%
5	University of Colorado Cancer Center	50%

**Figure 1:**
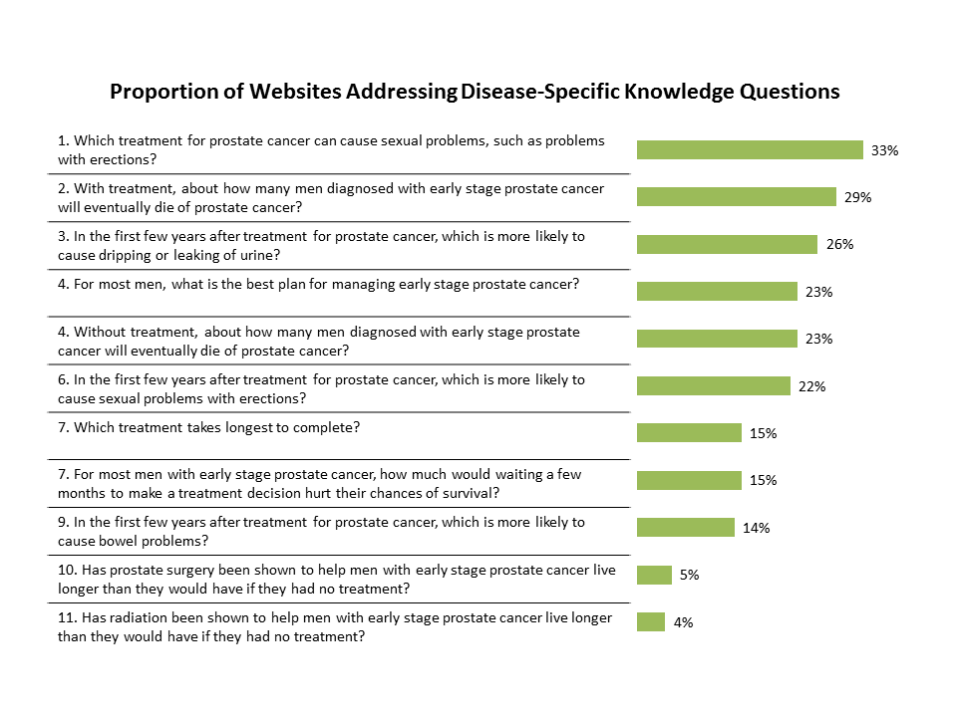
Proportion of NCI cancer center websites addressing early prostate cancer disease-specific knowledge questions.

**Figure 2:**
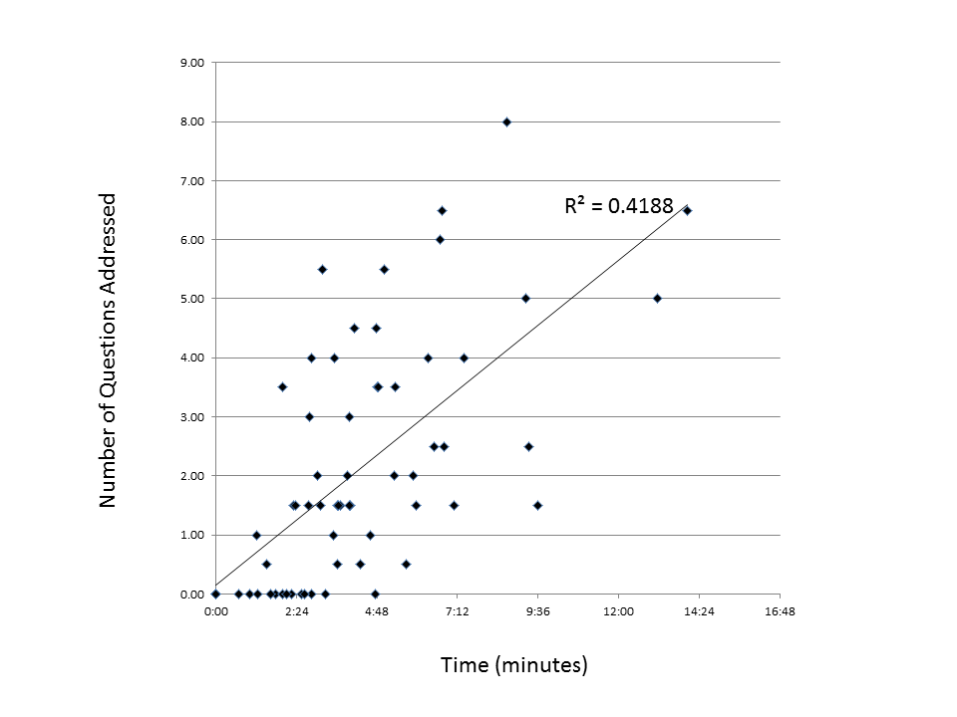
Relationship between website evaluation time and content measured by the number of early prostate cancer decision-specific knowledge questions addressed. Evaluation time was the time spent evaluating the website after reaching the prostate cancer-specific webpage. Websites with no prostate cancer-specific content (17%) were given evaluation times of 0 minutes.

Figure 1 lists the DQI questions in order of how frequently they were addressed. The question most commonly addressed by 33% of sites was, “Which treatment for prostate cancer can cause sexual problems, such as problems with erections?” The two questions least commonly addressed by 5% and 4% of sites, respectively, were, “Has radiation (or prostate surgery) been shown to help men with early prostate cancer live longer than they would have if they had no treatment?” Website evaluation time strongly correlated with the number of questions addressed (R^2^=0.42, p < 0.001) (Figure 2).

## Discussion

The majority of the 220,800 men diagnosed with prostate cancer have early stage disease and face a number of challenging and life-changing decisions about their treatment [5]. There are at least four broad categories of standard local therapy with similar efficacy and multiple emerging therapy options [2]. The internet is an important source of information that many men use to help make treatment decisions for prostate cancer [3]. In this study we evaluated the quality of information on cancer center websites using validated prostate cancer treatment decision-specific knowledge questions. These questions address important issues that patients should understand prior to making a treatment decision for prostate cancer. Using these questions as quality metrics, this study found that cancer center websites provide insufficient information to support decision making for men with early prostate cancer mainly due to a lack of information about and direct comparison of treatment outcomes and toxicity. On average, these websites provide only 19% of information deemed to be important in making an informed treatment decision. Furthermore, a significant proportion of websites do not have enough information about prostate cancer treatment options to answer any decision-specific knowledge questions. In a cohort of over 800 men having radical prostatectomy that completed the Early Prostate Cancer DQI, an average of 52% of the decision-specific knowledge questions were answered correctly [6]. Therefore, NCI cancer center websites contribute little, if any, additional knowledge to support shared decision making. However, we recognize the intended goal of many of these websites may not be to provide treatment information or support decision making. 

Important to note is the fact that our website evaluations addressed the presence of information relating to each DQI question, not necessarily the accuracy of that information. Reviewers were asked to determine if they felt there was adequate information available to answer each DQI question. The decision-specific knowledge questions in the Early Prostate Cancer DQI primarily focus on direct comparison of treatment outcomes or toxicity [4]. While many websites provide information on treatment toxicity and outcomes, this information is often discussed in isolation of each treatment modality with no direct comparisons. Therefore, the results of this study must be interpreted in the context of reviewer subjectivity. Both reviewers (CD and OB) were radiation oncology residents with knowledge of prostate cancer treatment. We found moderate inter-observer agreement based on the measures of percent agreement and the kappa coefficient. Agreement was likely limited by the open nature of the website evaluation instructions to determine if "adequate" information was available to address each DQI question. Another potential source of disagreement is the difference in thoroughness of website evaluation between reviewers, which will be discussed below. An ideal evaluation of website quality would involve actual patients using websites to complete the DQI questions and could be the subject of future investigation.

NCI-designated cancer center websites also have limited accessibility. Important treatment information is generally within multiple layers of content and often found in remote locations on the website. The time spent evaluating each website was strongly correlated with the number of questions addressed. This could suggest that sites with more content also had more important information. However, more content is not necessarily the most efficient or effective means of delivering important information. Important to consider is the potential inverse conclusion where longer evaluation time by the reviewer results in a greater number of questions addressed. While the reviewers sought to evaluate websites as thoroughly as possible, differences in the thoroughness of evaluation could explain discrepancies in inter-observer agreement. 

Prostate cancer treatment information is also less accessible to Spanish speaking patients and those using mobile devices. Only 24% of websites had a clear option to read information in Spanish. However, neither reviewer was fluent in Spanish. The quality of information in Spanish, if available, was not evaluated nor was the quality of translation from English to Spanish. We recognize that many web browsers offer the ability to translate website content to different languages. However, there is no oversight of the translation process by the organization providing the information when web browser based translation is used.

Limitations of this study include the subjective nature of website review. We sought to eliminate as much subjectivity as possible by using the DQI questions to evaluate information quality. However, some amount of subjectivity was necessary to judge whether sufficient information was available to answer each particular question. Inter-observer variability was moderate, likely due to the open nature of evaluation instructions and potential differences in the thoroughness of evaluation. Many websites have multiple layers of content and it is possible that we were unable to find information addressing some of the DQI questions. However, information that cannot be easily accessed on a website will likely not be helpful in aiding a man with prostate cancer in making a treatment decision.

## Conclusions

The NCI-designated cancer centers represent the premier cancer research and treatment centers in the United States, and their websites are an important, trusted source of information that patients utilize in making treatment decisions. Overall, these websites are inadequate sources of information to support decision making for prostate cancer patients. However providing treatment information and supporting decision making may not be the intended goal of many of these institutional websites. Potential changes to improve the quality and accessibility of prostate cancer treatment information include expanding Spanish and mobile device accessibility and providing data on the benefits, outcomes, and toxicity of each specific treatment in formats such as tables and charts that allow for direct comparison.
